# MAP-SCTNet: multi-scale pyramid and frequency-enhanced network for colorectal cancer histopathological image segmentation

**DOI:** 10.3389/fmed.2026.1760720

**Published:** 2026-03-13

**Authors:** Haitao Chen, Zhengxu Duan, Lan Li, Jie Wei, Jia Zhang, Rongchao Zheng

**Affiliations:** 1 Chengdu Qingbaijiang District People's Hospital, Chengdu, China; 2 Yibin Research Institute of University of Electronic Science and Technology of China, Yibin, China; 3 School of Optoelectronic Science and Engineering, University of Electronic Science and Technology of China, Chengdu, China

**Keywords:** dual-domain enhancement, frequency-texture enhancement, image segmentation, knowledge distillation, multi-scale feature extraction

## Abstract

Medical image segmentation remains challenging due to the morphological heterogeneity of lesions, inherently low contrast with ambiguous boundaries, and limited availability of annotated training data. Existing methods often struggle to simultaneously capture multi-scale contextual information and preserve fine-grained boundary details, particularly in resource-constrained clinical settings. To address these challenges, we propose MAP-SCTNet, an efficient segmentation network that enhances the SCTNet architecture through three synergistic innovations. First, we design a Multi-Scale Atrous Spatial Pyramid Pooling (MS-ASPP) module that employs five parallel dilated convolution branches coupled with a dual-attention mechanism and boundary-aware refinement, enabling comprehensive multi-scale feature extraction while maintaining precise edge localization. Second, we introduce an Adaptive Frequency Enhancement and Texture-Aware Module (AFE-TAM) that operates in the frequency domain to adaptively fuse low, mid, and high-frequency components, complemented by Gabor filtering and Local Binary Patterns for robust texture representation across pathological subtypes. Third, we develop a Progressive Dual-Teacher Knowledge Distillation (PDTKD) framework that leverages both Transformer-based and CNN-based teacher networks through a three-stage training strategy, effectively bridging the gap between training and inference while improving generalization from limited data. Extensive experiments on the EBHI-SEG colorectal cancer histopathological dataset, which comprises biopsy specimens collected via colonoscopy, demonstrate that MAP-SCTNet achieves state-of-the-art performance with a mean Dice coefficient of 83.76% and mean IoU of 74.5%, surpassing recent methods including TransUNet, Swin-UNet, and SegNeXt. Notably, our model requires only 21.8M parameters and 30.7G FLOPs, representing a 79.3% reduction in parameters and 71.1% reduction in computation compared to TransUNet, making it well-suited for real-time computer-aided diagnosis in clinical colorectal cancer diagnosis workflows.

## 1 Introduction

Colorectal cancer remains one of the most prevalent malignancies worldwide, encompassing both colon and rectal cancers. According to the American Cancer Society's 2024 statistics, colorectal cancer incidence among adults aged 30–44 has increased by 1%−2% annually, rising from the fourth leading cause of cancer death in individuals under 50 during the late 1990s to now ranking first in men and second in women (1). Despite these alarming trends, colorectal cancer is among the few cancers that can be effectively prevented through early screening and detection (2, 3). In clinical practice, pathologists examine H&E-stained tissue sections under microscopy to identify and localize lesions (4, 5). However, this conventional approach heavily relies on subjective physician experience and lacks standardized quantitative assessment, highlighting the urgent need for computer-aided diagnostic tools to ensure consistent and accurate histological diagnosis (6).

Automated segmentation of colorectal lesions in endoscopic and histopathological images faces three fundamental challenges that remain inadequately addressed. First, multi-scale morphological heterogeneity: colorectal lesions exhibit substantial variations in size, shape, and appearance across different pathological stages—from millimeter-scale early polyps to large irregular advanced tumors—demanding models capable of simultaneously capturing fine-grained local details and broad contextual information (7, 8). Second, inherently low contrast and ambiguous boundaries: unlike natural images with well-defined edges, medical images suffer from non-uniform illumination, similar intensity distributions between lesions and surrounding tissues, and complex textural patterns, making precise boundary delineation extremely challenging even for experienced clinicians (9, 10). Third, limited annotated data and generalization difficulty: pixel-level annotation of medical images requires specialized expertise and is prohibitively time-consuming, resulting in relatively small training datasets that pose significant challenges for deep learning models to generalize across diverse patients, imaging devices, and clinical centers (11, 12).

Existing deep learning approaches have attempted to address these challenges with varying degrees of success, yet significant limitations persist. CNN-based architectures such as U-Net and its variants (13, 14) excel at extracting local features and fine details through hierarchical convolutions, but their inherently limited receptive fields make it difficult to model long-range dependencies essential for understanding the global context of morphologically diverse lesions. Transformer-based methods including TransUNet and Swin-UNet effectively capture global semantic relationships through self-attention mechanisms, but incur substantial computational overhead—TransUNet requires 105.3M parameters and 106.3G FLOPs—limiting their practical deployment in resource-constrained clinical environments. Moreover, the pursuit of global modeling often sacrifices boundary precision, resulting in inadequate delineation of lesions with ambiguous edges. Lightweight segmentation networks designed for real-time applications typically achieve efficiency at the cost of accuracy, particularly struggling with challenging cases involving small lesions or fuzzy boundaries. Furthermore, most existing methods operate exclusively in the spatial domain, neglecting frequency-domain information that could provide complementary cues for boundary enhancement and texture discrimination in low-contrast medical images. The training-inference inconsistency inherent in knowledge distillation frameworks—where student networks trained with teacher guidance must perform independently during deployment—also remains underexplored, limiting the effectiveness of knowledge transfer for improving small-sample generalization.

Recently, SCTNet (15) has emerged as a promising architecture that cleverly integrates CNN's local detail extraction capabilities with Transformer's global semantic understanding through a dual-branch training and single-branch inference design, partially alleviating the trade-off between accuracy and efficiency. However, SCTNet was originally designed for natural scene parsing and exhibits several limitations when applied to medical image segmentation: (1) insufficient multi-scale feature extraction for handling the extreme size variations of colorectal lesions; (2) lack of mechanisms to enhance low-contrast boundaries and exploit texture information characteristic of medical images; and (3) suboptimal knowledge transfer that does not fully address training-inference inconsistency or leverage domain-specific prior knowledge.

- To address multi-scale morphological heterogeneity, we design a Multi-Scale Atrous Spatial Pyramid Pooling (MS-ASPP) module inserted at H/8 × W/8 resolution. This module employs five parallel branches with varying dilation rates (1 × 1 convolution, 3 × 3 convolution, atrous convolutions with rates of 6, 12, and 18, and global average pooling), coupled with a dual-attention mechanism (channel and spatial) and a boundary-aware sub-module. This design enables comprehensive multi-level feature capture from local to global scales while maintaining precise edge localization, significantly improving recognition of small and medium-sized lesions.
- To overcome low contrast and ambiguous boundaries, we introduce an Adaptive Frequency Enhancement and Texture-Aware Module (AFE-TAM) embedded at H/16 × W/16 resolution. This module leverages Fourier transform to decompose images into low, mid, and high-frequency components with learnable adaptive weighting, complemented by multi-scale texture extraction through Gabor filter banks (8 orientations × 4 scales) and Local Binary Patterns (LBP). A category-adaptive detector dynamically adjusts frequency processing strategies based on the detected pathological subtype, optimizing enhancement for each of the six colorectal lesion categories;
- To improve generalization with limited annotated data, we develop a Progressive Dual-Teacher Knowledge Distillation (PDTKD) framework that leverages complementary knowledge from two teacher networks: the original Transformer branch capturing global semantics and a pre-trained ResNet-UNet providing domain-relevant structural priors. A three-stage training strategy (joint dual-teacher distillation for the first 40% epochs, dynamic weight adjustment for the middle 40%, and student autonomous learning for the final 20%) explicitly addresses training-inference inconsistency while enhancing small-sample generalization capability.

Extensive experiments on the EBHI-SEG colorectal cancer histopathological image dataset (16) demonstrate that MAP-SCTNet achieves state-of-the-art segmentation performance while maintaining computational efficiency suitable for clinical deployment. The remainder of this paper is organized as follows: Section 2 reviews related work; Section 3 details the proposed methodology; Section 4 presents experimental results and analysis; and Section 5 concludes the paper.

## 2 Related work

Medical image segmentation plays an important role in computer vision and medical diagnosis. Fully Convolutional Network (FCN) and U-Net are two representative models that have laid the foundation for this field (17). Guo X et al. (18) used FCN for the task of segmentation of liver and tumors in CT images. Bi et al. (19) proposed Stacked-FCN using multi-channel learning and cascaded fully convolutional networks for more accurate and robust medical image segmentation. U-Net (20) further incorporates skip connections to transfer information between features at different levels, effectively capturing boundary information and significantly improving segmentation accuracy. Xiao et al. (21) proposed Res-UNet for retinal blood vessel segmentation, introducing residual connections to preserve fine vessel information. Alom et al. (22) proposed R2U-Net using recurrent residual convolutional layers to enhance contextual information extraction. Li et al. (23) proposed H-DenseUNet for liver and tumor 3D image segmentation through hybrid dense connectivity. More recently, Isensee et al. (24) introduced nnU-Net, a self-configuring framework that automatically adapts preprocessing, architecture, and training strategies to specific datasets, establishing a strong baseline that has dominated numerous medical image segmentation challenges. However, CNN networks excel at capturing local features but lack global feature representations due to their limited receptive fields, making it difficult to comprehensively capture global contextual information.

In 2017, the Transformer network proposed by Vaswani et al. (25) excelled in learning long-range feature representations. TransUNet (26) and TransSegNet (27) combine U-Net with Transformer to enhance both local and global feature processing. Swin Transformer (28) introduces a hierarchical structure for multi-scale feature modeling, while Swin-Unet (29) combines Swin Transformer with U-Net, demonstrating strong performance in handling fuzzy boundaries and low contrast regions. However, these Transformer-based methods typically require substantial computational resources, limiting their deployment in resource-constrained clinical environments.

Recent advances in polyp and medical image segmentation have introduced several innovative architectures. Fan et al. (8) proposed PraNet, which employs parallel reverse attention to aggregate features and achieve accurate polyp boundary detection. Dong et al. (30) introduced Polyp-PVT, utilizing Pyramid Vision Transformer as the encoder with a cascaded fusion module and camouflage identification module to effectively suppress noise and improve feature expressiveness for polyp segmentation. Wang et al. (31) proposed SSFormer, which uses a pyramid Transformer encoder with a stepwise feature aggregation decoder to progressively fuse multi-level features, improving generalization on unseen colonoscopy data. Duc et al. (32) developed ColonFormer, a lightweight Transformer-based encoder-decoder architecture capable of modeling long-range semantic information at multiple scales for efficient colon polyp segmentation. Valanarasu et al. (33) proposed UNeXt, an MLP-based architecture that reduces parameters by 72× and computational complexity by 68 × compared to TransUNet while maintaining competitive segmentation performance, demonstrating the potential of efficient architectures for point-of-care applications.

Beyond polyp segmentation, recent studies have further advanced the broader landscape of endoscopic and colorectal cancer image analysis. Narasimha Raju et al. (34) proposed a synergistic AI framework that integrates hyper-granular image dissection with precision segmentation and automated diagnosis for colorectal cancer, demonstrating the effectiveness of combining fine-grained feature extraction with end-to-end diagnostic pipelines. Podda et al. (35) developed a deep learning strategy for 3D segmentation of colorectal tumors from ultrasound imaging, extending the applicability of deep learning-based segmentation beyond conventional endoscopic modalities to volumetric ultrasound data. Liu et al. (36) proposed GLBFormer, a Global-to-Local Deep Interaction and Boundary-Aware Transformer for accurate polyp segmentation, which leverages multi-scale global-to-local interaction mechanisms to achieve improved boundary delineation in complex endoscopic scenes. Furthermore, in the related domain of medical image segmentation, Li et al. (37) proposed a deep feature fusion approach for acute ischemic stroke lesion segmentation, demonstrating that multi-level feature fusion strategies can effectively capture lesion boundaries in challenging imaging conditions, an insight that is also applicable to endoscopic image analysis. These studies collectively highlight the growing research interest in developing more accurate and robust segmentation methods for colorectal cancer diagnosis, motivating the need for architectures that can effectively handle the unique challenges of endoscopic images, including low contrast, blurred boundaries, and variable lesion morphology.

The emergence of foundation models has opened new paradigms for medical image segmentation. Ma et al. (38) introduced MedSAM, adapting the Segment Anything Model (SAM) for universal medical image segmentation by fine-tuning on a large-scale dataset of 1.57 million image-mask pairs covering 10 imaging modalities and over 30 cancer types. While MedSAM demonstrates impressive generalization capabilities, it requires interactive prompts (bounding boxes) during inference and incurs substantial computational overhead, limiting its applicability for fully automated clinical workflows.

To further balance the local efficiency of CNN with the global modeling capability of Transformer, SCTNet proposes a hybrid architecture that introduces Transformer branches during training to capture long-range dependencies while retaining only the lightweight CNN backbone during inference, achieving efficient single-branch inference. This model has demonstrated significant advantages in 2D medical image segmentation tasks. However, SCTNet still suffers from insufficient multi-scale feature extraction, resulting in limited adaptability to lesions of different sizes; training-inference inconsistency makes it difficult to fully transfer knowledge from the auxiliary Transformer branch to the CNN backbone; and the adaptability to medical image characteristics is poor, making it difficult to effectively handle challenges such as low contrast, blurred boundaries, and complex textures.

## 3 Materials and methods

Taking inspiration from references (39–41), this paper proposes an improved SCTNet network architecture, abbreviated as MAP-SCTNet, to address the issues of insufficient multi-scale feature extraction, inconsistent training inference, and poor adaptability of medical image characteristics in 2D medical image segmentation tasks of the original SCTNet. The network maintains the efficient single branch inference structure of the original SCTNet, while introducing three innovatively designed modules at key locations to comprehensively improve the performance of medical image segmentation.

The core innovation of MAP-SCTNet lies in systematically addressing three critical bottlenecks in colorectal cancer endoscopic image segmentation: (1) insufficient multi-scale feature representation for lesions with diverse morphologies, (2) inadequate enhancement of low-contrast boundaries and complex textures inherent to medical images, and (3) training-inference inconsistency limiting model generalization. Unlike existing methods that address these challenges in isolation, our approach integrates three synergistic modules—MS-ASPP for hierarchical multi-scale feature capture, AFE-TAM for dual-domain frequency-texture enhancement, and PDTKD for progressive knowledge transfer—forming a unified framework that achieves superior segmentation performance while maintaining computational efficiency suitable for clinical deployment.

**Figure 1:**
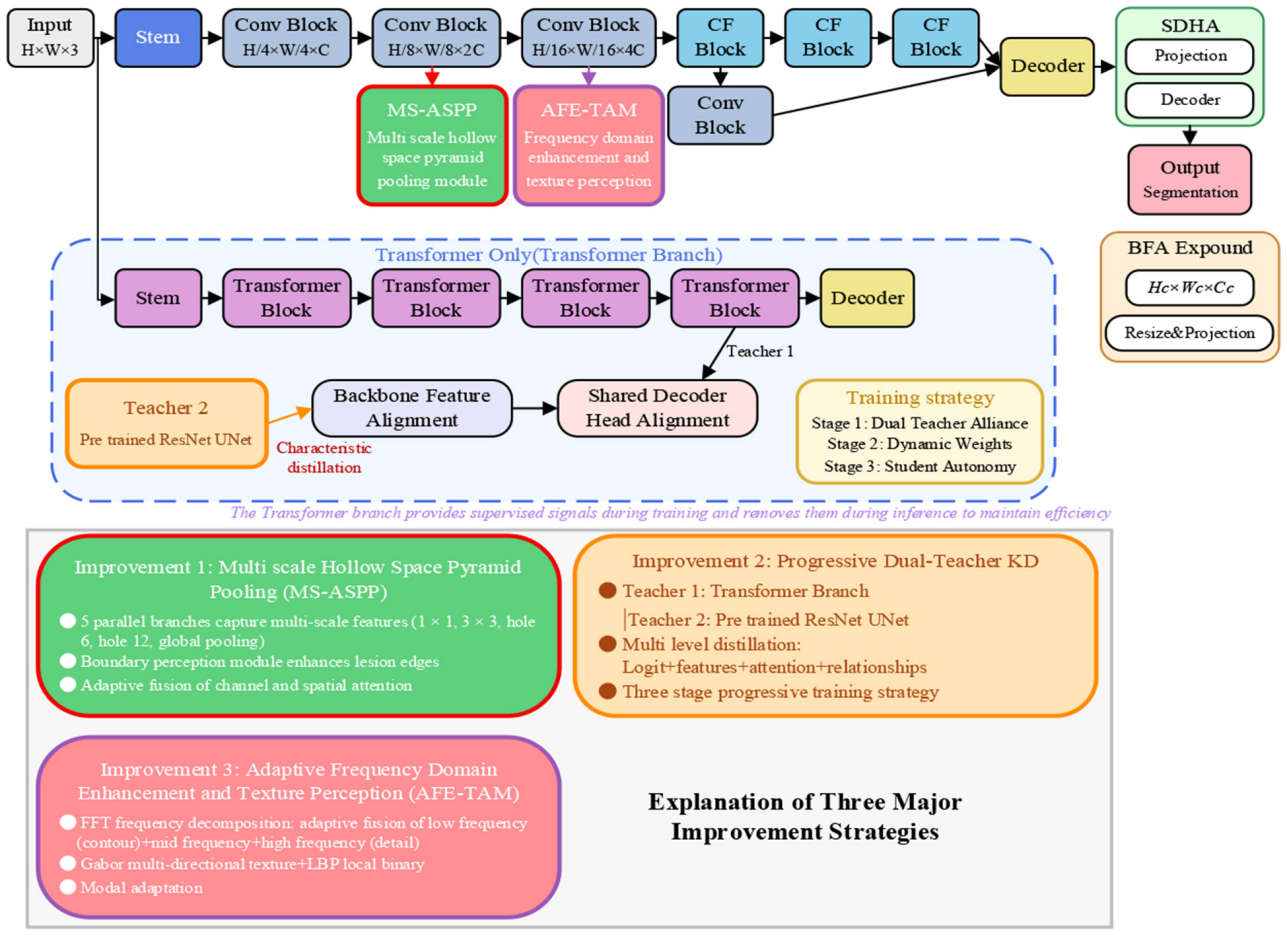
The overall architecture of the MAP-SCTNet network framework. Composed of MS-ASPP, PDTKD, and AFE-TAM.

The overall architecture of MAP-SCTNet is shown in Figure 1. The network adopts an encoder decoder structure. The encoder first extracts initial features from the input image through the Stem module, and then gradually extracts deep features and reduces spatial resolution through multiple convolutional blocks. Unlike the original SCTNet, this paper inserts two enhancement modules at key positions in the encoder:

MAP-SCTNet incorporates two enhancement modules at different resolution levels of the backbone: the MS-ASPP module is embedded after the H/8 × W/8 × 2C layer, capturing multi-scale features through five parallel branches (including 1 × 1 convolution, 3 × 3 convolution, two dilated convolutions with different dilation rates, and global average pooling) with a dual attention mechanism; the AFE-TAM module is embedded after the H/16 × W/16 × 4C layer, decomposing features into low, mid, and high-frequency bands via Fourier transform with adaptive fusion, while extracting multi-scale and multi-directional texture information using Gabor filter banks (8 directions × 4 scales) and Local Binary Patterns (LBP). During training, the PDTKD framework retains the Transformer branch of the original SCTNet as Teacher 1 and introduces ImageNet pre-trained ResNet-UNet as Teacher 2, enabling the CNN student network to simultaneously learn the global semantic understanding of Transformers and the prior knowledge of pre-trained models through a three-stage progressive training strategy (dual-teacher joint distillation for 40% epochs, dynamic weight adjustment for 40% epochs, and student self-learning for 20% epochs). During inference, only the lightweight CNN backbone with MS-ASPP and AFE-TAM modules is retained, ensuring real-time inference performance.

Below, we will provide a detailed introduction to the design principles, mathematical derivation, and implementation details of the three core improvement modules.

### 3.1 Multi-scale atrous spatial pyramid pooling

**Figure 2:**
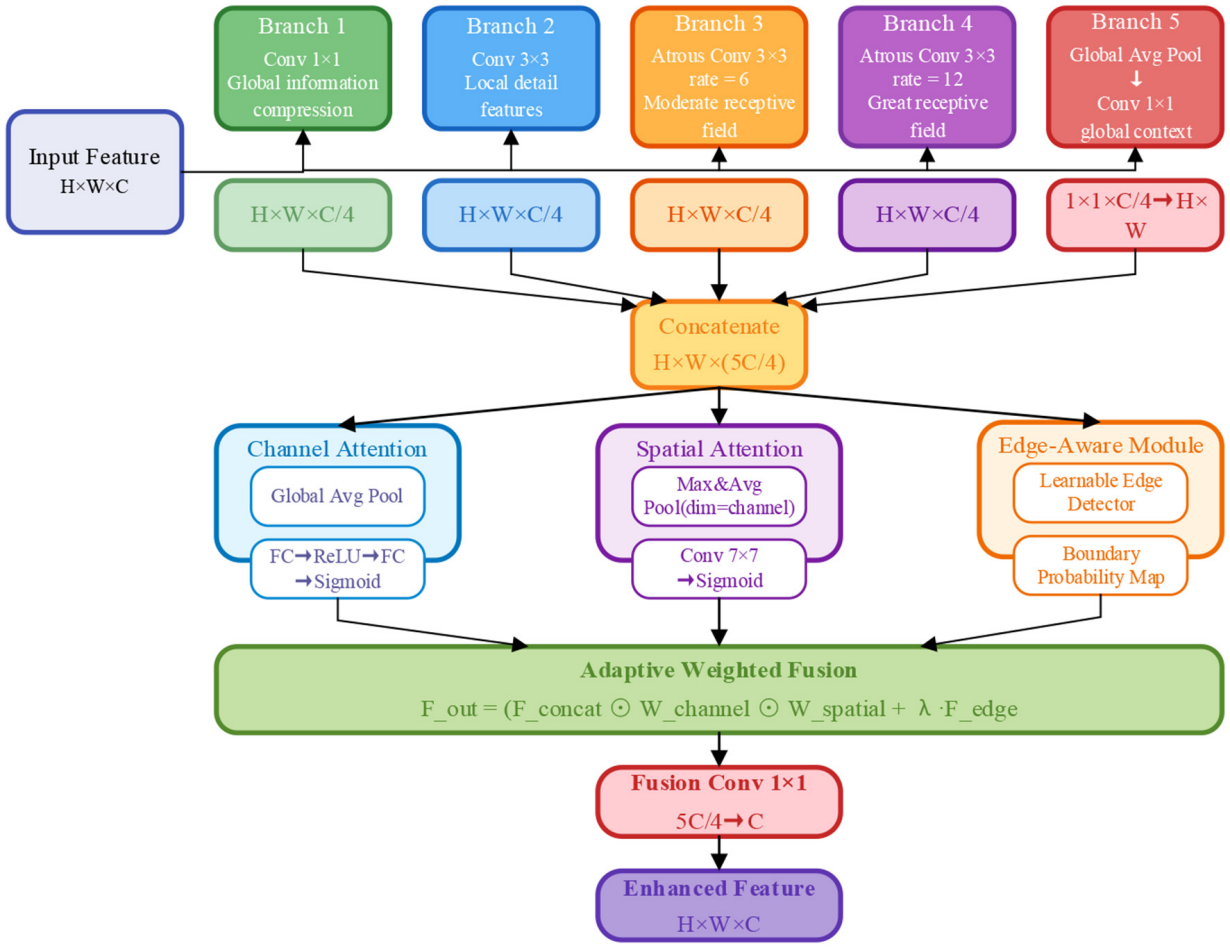
Module structure diagram of MS-ASPP.

To effectively capture lesion features at different scales in 2D medical images, this paper proposes the Multi-Scale Atrous Spatial Pyramid Pooling (MS-ASPP). Through parallel multi-branch architecture and adaptive attention mechanisms, this module can simultaneously extract multi-level features from local details to global context. The structure of the MS-ASPP module is shown in Figure 2.

As shown in Figure 2, the MS-ASPP module consists of three core components: five parallel branches, dual attention mechanism, and boundary perception module. Given the input feature map $F_{in}\in R^{H\times W\times C}$, where *H* and *W* represent the height and width of the feature map, and *C* represents the number of channels, the complete processing flow of this module can be represented as:

#### 3.1.1 Multi-scale parallel feature extraction

Given an input feature map $F_{in}\in R^{H\times W\times C}$, where *H* and *W* represent the height and width of the feature map, and *C* denotes the number of channels, the five parallel branches of MS-ASPP are responsible for capturing feature information at different scales:

Branch 1 (1 × 1 convolution) performs channel compression through 1 × 1 convolution to capture global pixel-level information:


$$\begin{array}{l} F_{1}=Conv_{1\times 1}\left(F_{in}\right)\in R^{H\times W\times C/4} \end{array}$$ (1)

Branch 2 (3 × 3 convolution): standard 3 × 3 convolution extracts local neighborhood features while preserving spatial detail information:


$$\begin{array}{l} F_{2}=Conv_{3\times 3}\left(F_{in}\right)\in R^{H\times W\times C/4} \end{array}$$ (2)

Branch 3 and branch 4 (atrous convolution): using 3 × 3 atrous convolution with different atrous rate, the receptive field is extended to 13 × 13 (rate = 6) and 25 × 25 (rate = 12), respectively:


$$\begin{array}{l} F_{3}=AtrousConv_{3\times 3}^{r=6}\left(F_{in}\right),\;F_{4}=AtrousConv_{3\times 3}^{r=12}\left(F_{in}\right) \end{array}$$ (3)

The effective receptive field of atrous convolution is calculated as *RF* = *k* + (*k* − 1) × (*r* − 1), where *k*=3 is the kernel size and *r* is the dilation rate. This design enables the module to effectively capture medium-scale lesion features (rate = 6) and large-scale organ contours (rate = 12) while maintaining parameter efficiency.

**Branch 5 (global average pooling)** encodes global contextual information through global average pooling and 1 × 1 convolution, then upsamples to the original resolution:


$$\begin{array}{l} F_{5}=Upsample\left(Conv_{1\times 1}\left(GAP\left(F_{in}\right)\right)\right)\in R^{H\times W\times C/4} \end{array}$$ (4)

The features extracted from the five branches are concatenated along the channel dimension to form a feature representation rich in multi-scale information:


$$\begin{array}{l} F_{concat}=Concat\left(\left[F_{1},F_{2},F_{3},F_{4},F_{5}\right]\right)\in R^{H\times W\times 5C/4} \end{array}$$ (5)

#### 3.1.2 Dual adaptive attention mechanism

To achieve adaptive weighted fusion of multi-scale features, this paper designs a dual attention mechanism combining channel attention and spatial attention.

Channel Attention: applies global average pooling to the concatenated features *F*_*concat*_, then generates channel attention weights through two fully connected layers:


Wc=σ(FC2(ReLU(FC1(GAP(Fconcat))))) (6)

where $FC_{1}:R^{5C/4}\to R^{5C/32}$ and $FC_{2}:R^{5C/32}\to R^{5C/4}$ represent two fully connected layers, and σ(·) denotes the Sigmoid function. The channel attention weights $W_{c}\in R^{1\times 1\times 5C/4}$ can adaptively adjust the importance of different feature channels.

Spatial attention applies max pooling and average pooling to *F*_*concat*_ along the channel dimension, concatenates the two pooling results, and generates spatial attention weights through 7 × 7 convolution:


$$\begin{array}{l} W_{s}=\sigma \left(Conv_{7\times 7}\left(\left[MaxPool_{c}\left(F_{concat}\right);AvgPool_{c}\left(F_{concat}\right)\right]\right)\right) \end{array}$$ (7)

where $W_{s}\in R^{H\times W\times 1}$ can emphasize critical regions in the spatial dimension. The weighted features are represented as:


$$\begin{array}{l} F_{attn}=F_{concat}⊙W_{c}⊙W_{s} \end{array}$$ (8)

where ⊙ denotes element-wise multiplication (Hadamard product). This dual attention mechanism enables the module to adaptively select the most important features in both channel and spatial dimensions.

#### 3.1.3 Edge-aware module

Precise boundary localization is crucial in medical image segmentation tasks. To this end, this paper designs a boundary-aware module to explicitly extract boundary features. The module generates a boundary probability map through a learnable edge detection operator:


$$\begin{array}{l} F_{edge}=Conv_{3\times 3}\left(EdgeDetector\left(F_{in}\right)\right) \end{array}$$ (9)

where EdgeDetector (·) is a learnable edge detector implemented with 3 × 3 depthwise separable convolution. The term “learnable” indicates that the convolutional kernel weights are trainable parameters updated via backpropagation during end-to-end training, rather than fixed hand-crafted operators such as Sobel or Laplacian filters. The gradient signal flows from the segmentation loss through *F*_*edge*_ to continuously optimize the detector for colorectal histopathological image boundaries. Finally, the attention-weighted multi-scale features and boundary features are combined through adaptive weighted fusion:


$$\begin{array}{l} F_{fused}=F_{attn}+\lambda \cdot F_{edge} \end{array}$$ (10)

where λ is a learnable weight parameter, initialized to 0.3. The fused features are adjusted back to *C* channels through 1 × 1 convolution:


$$\begin{array}{l} F_{out}=Conv_{1\times 1}\left(F_{fused}\right)\in R^{H\times W\times C} \end{array}$$ (11)

The entire MS-ASPP module adds only 2.3M parameters and 1.8G FLOPs by using depthwise separable convolutions instead of standard convolutions, significantly enhancing multi-scale feature extraction capability while maintaining computational efficiency.

Compared to conventional atrous spatial pyramid pooling methods that employ fixed dilation rates, MS-ASPP introduces an edge-aware sub-module and dual attention mechanism, which explicitly model boundary characteristics and adaptively weight multi-scale features. This design addresses the fundamental limitation of existing approaches in capturing precise lesion boundaries, particularly for small-to-medium sized polyps and serrated adenomas with irregular morphologies. Ablation studies (Section 4.4) demonstrate that MS-ASPP contributes a 4.73% improvement in average Dice coefficient, validating its critical role in multi-scale lesion recognition.

### 3.2 Progressive dual-teacher KD framework for knowledge distillation

**Figure 3:**
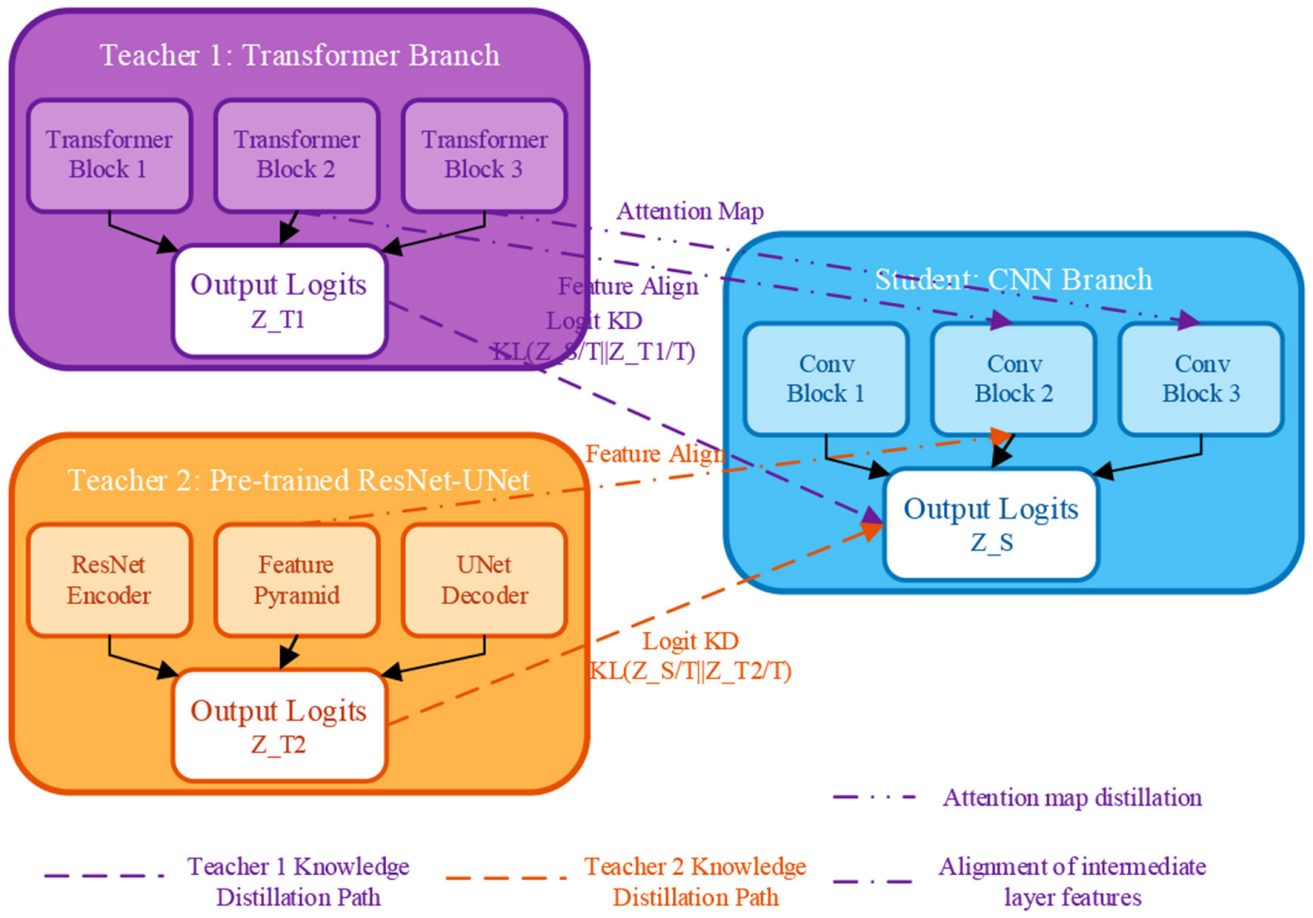
Progressive dual teacher knowledge distillation, PDTKD structure diagram.

To address the training-inference inconsistency issue in the original SCTNet—where the Transformer branch used during training is discarded at inference—and to fully leverage external pre-trained knowledge for improved generalization, this paper proposes the Progressive Dual-Teacher Knowledge Distillation (PDTKD) framework. Traditional single-teacher distillation suffers from knowledge redundancy and limited diversity. PDTKD overcomes this by introducing two complementary teachers: (1) teacher 1 (Transformer branch) provides global semantic understanding through long-range dependency modeling, and (2) teacher 2 (ImageNet pre-trained ResNet-UNet) contributes rich low-level visual priors and robust feature representations. Through multi-level distillation (logit, feature, attention, and relation) and a three-stage progressive training strategy, PDTKD enables the student CNN network to simultaneously inherit global context awareness and pre-trained domain knowledge, while gradually achieving autonomous learning capability. This design fundamentally resolves the gap between training and inference, significantly enhancing model robustness on small-sample and hard-sample scenarios. The structure of the PDTKD framework is shown in Figure 3.

As shown in Figure 3, the PDTKD framework consists of two teacher networks, one student network, and a multi-level distillation path.

#### 3.2.1 Dual teacher network architecture

**Teacher 1 (T1)—Transformer Branch** retains the original SCTNet's Transformer branch as the first teacher, which excels at capturing long-range dependencies and global semantic information. Given input *X* ∈ *R*^*H* × *W* × 3^, the output logits of the Transformer branch are represented as *Z*_*T*1_ = *f*_*T*1_(*X*; θ_*T*1_).

**Teacher 2 (T2)—Pre-trained ResNet-UNet** introduces a ResNet-UNet pre-trained on ImageNet as the second teacher, which possesses strong feature extraction capability and good generalization performance. Its output is represented as *Z*_*T*2_ = *f*_*T*2_(*X*; θ_*T*2_).

**Student network (S)—CNN branch** refers to the CNN backbone of SCTNet as the student network, which needs to learn knowledge from both teachers. Its output is represented as *Z*_*S*_ = *f*_*S*_(*X*; θ_*S*_).

#### 3.2.2 Multi level knowledge distillation

PDTKD adopts four complementary distillation strategies to convey teacher knowledge from different levels:

**(1) Logit distillation (output layer)** uses KL divergence to constrain the consistency between student and teacher output distributions:


$$\begin{array}{l} L_{\log it}^{i}=KL\left(soft\max\left(\frac{Z_{S}}{T}\right)\|soft\max\left(\frac{Z_{Ti}}{T}\right)\right) \end{array}$$ (12)

where *T* is the temperature parameter used to soften the probability distribution. For medical image segmentation tasks, this paper sets *T* = 4.

**(2) Feature distillation (intermediate layers)** aligns intermediate layer features at 1/4, 1/8, and 1/16 resolutions of the encoder. Let $F_{S}^{l}$ be the student features at layer *l* and $F_{T_{i}}^{l}$ be the teacher features. First, use 1 × 1 convolution for channel alignment $\overset{\sim }{F_{S}^{l}}=Conv_{1\times 1}\left(F_{S}^{l}\right)$. The feature distillation loss is:


$$\begin{array}{l} L_{feat}^{i}=\underset{l\in \left\{1/4,1/8,1/16\right\}}{\sum }\|\overset{\sim }{F_{S}^{l}-F_{Ti}^{l}}{\|}_{2}^{2} \end{array}$$ (13)

**(3) Attention distillation (critical regions)** extracts the self-attention map $A_{T1}\in R^{N\times N}$ from the Transformer teacher (*N* = *HW* is the number of spatial positions) to guide the CNN student to learn similar attention patterns. The student's attention map is obtained by normalizing feature maps and computing the similarity matrix:


$$\begin{array}{l} A_{S}=soft\max\left(\frac{F_{S}{\left(F_{S}\right)}^{T}}{\sqrt{d}}\right) \end{array}$$ (14)

The attention distillation loss employs the Frobenius norm: $L_{attn}=\|A_{S}-A_{T1}{\|}_{F}^{2}$.

**(4) Relation distillation (inter-sample relations)** computes the similarity matrix between samples within each batch *R*_*Ti*_(*j, k*) = *sim*(*f*_*Ti*_(*X*_*j*_), *f*_*Ti*_(*X*_*k*_)), *j, k* ∈ {1, ..., *B*} to enable the student to learn the teacher's modeling of different sample relationships. The relation distillation loss is $L_{rel}^{i}=\|R_{S}-R_{Ti}{\|}_{F}^{2}$.

The complete distillation loss from teacher *i* is:


$$\begin{array}{l} L_{KD}^{i}=L_{\log it}^{i}+\gamma \cdot L_{feat}^{i}+\delta \cdot L_{attn}^{i}+\eta \cdot L_{rel}^{i} \end{array}$$ (15)

According to experimental validation, we set γ = 0.3, δ = 0.2 (only for T1), and η = 0.1.

#### 3.2.3 Three stage progressive training strategy

In order to avoid training inference differences and enable student to gradually become independent, this paper designs a three-stage progressive training strategy:

**Stage 1: dual-teacher joint distillation (first 40% epochs)**

In the early training phase, the student learns from both teachers simultaneously. The total loss function is:


$$\begin{array}{l} L_{stage1}=L_{seg}+\alpha \cdot L_{KD}^{1}+\beta \cdot L_{KD}^{2} \end{array}$$ (16)

where $L_{seg}$ is the standard segmentation loss (combination of Dice Loss and Focal Loss). Based on experiments, we set α = 0.5 and γ = 0.3.

**Stage 2: dynamic teacher weight adjustment (middle 40% epochs)**

In the second stage, the weights of the two teachers are dynamically adjusted based on validation set performance. Every *K* epochs (we set *K* = 5), the weights are updated according to the student's performance on the validation set after teacher guidance:


$$\begin{array}{l} \{\begin{array}{l} \alpha \leftarrow \alpha +0.1,\;\beta \leftarrow \beta -0.05,\;if\;Dice_{T1}>Dice_{T2}\\ \alpha \leftarrow \alpha -0.05,\;\beta \leftarrow \beta +0.1,\;otherwise \end{array} \end{array}$$ (17)

Meanwhile, a Curriculum Learning strategy is introduced, where sample difficulty is defined as the reciprocal of its Dice coefficient. The top *p*% difficult samples receive an additional weight *w*_*hard*_ = 2.0 in the loss function:


$$\begin{array}{l} L_{stage2}=L_{seg}+\alpha \left(t\right)\cdot L_{KD}^{1}+\beta \left(t\right)\cdot L_{KD}^{2}+w_{hard}\cdot L_{hard} \end{array}$$ (18)

where the proportion of difficult samples *p* grows linearly from 20 to 50%.

**Stage 3: student self-learning (last 20% epochs)** In the late training phase, teacher branches are randomly dropped out to promote student independent learning. At each training step, teacher supervision is removed with probability *p*_*drop*_ = 0.5:


$$\begin{array}{l} L_{stage3}=\{\begin{array}{l} L_{seg}\;with\;probability\;p_{drop}\\ L_{seg}+\alpha \cdot L_{KD,hard}^{1}+\beta \cdot L_{KD,hard}^{2},\;otherwise \end{array} \end{array}$$ (19)

where $L_{KD,hard}^{1}$ denotes the distillation loss computed only on difficult samples. This strategy ensures that the student network can smoothly transition to a completely independent inference phase.

The three-stage progressive strategy is designed to balance knowledge absorption and model independence. Stage 1 establishes a solid foundation through joint supervision from both teachers; Stage 2 dynamically adjusts teacher influence based on validation performance, preventing bias toward a single teacher while introducing curriculum learning to prioritize hard samples; Stage 3 promotes student autonomy by randomly dropping teacher guidance, ensuring the student network can generalize independently during inference. This progressive paradigm contrasts with conventional static distillation schemes, offering superior adaptability to diverse lesion types and limited training data—critical factors in clinical medical image analysis.

#### 3.2.4 Difficult sample mining and consistency constraints

To further enhance generalization capability in small-sample scenarios, this paper introduces hard sample mining and data augmentation consistency regularization. For sample *i*, if it satisfies *Dice*_*i*_ < 0.7 or HD95_*i*_ > 20, it is marked as a difficult sample and receives higher weight in loss computation.

Data augmentation consistency regularization applies different augmentations to the same image and constrains the student's predictions on augmented samples to remain consistent:


$$\begin{array}{l} L_{consist}=\frac{1}{N_{aug}}\overset{N_{aug}}{\underset{j=1}{\sum }}\|P_{S}\left(Aug_{j}\left(X\right)\right)-P_{S}\left(X\right){\|}_{2}^{2} \end{array}$$ (20)

where *Aug*_*j*_(·) represents the *j*-th augmentation operation, and *P*_*S*_(·) denotes the student network's prediction probability. This constraint is added to the total loss in stages 2 and 3 with a weight of 0.1.

Through the PDTKD framework, the student network can operate completely independently during inference while acquiring both the Transformer's global semantic understanding and the pre-trained model's rich prior knowledge.

### 3.3 Adaptive frequency enhancement and texture-aware module

**Figure 4:**
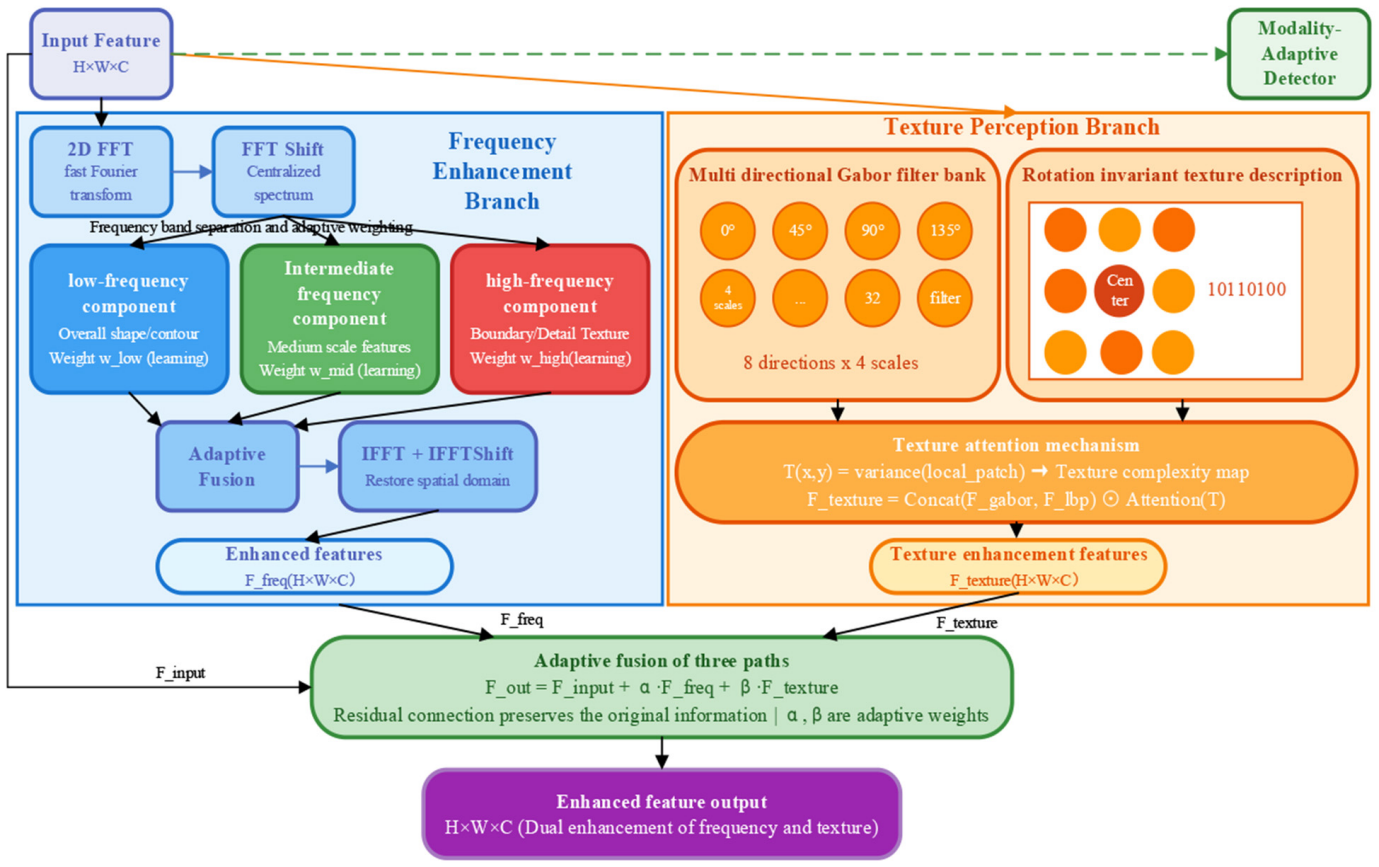
AFE-TAM module structure diagram.

2D medical images commonly suffer from issues such as low contrast, blurred boundaries, and complex textures, which traditional spatial-domain convolution struggles to effectively address. Frequency-domain analysis can decompose images into components representing global structure (low-frequency) and fine details (high-frequency), enabling targeted enhancement. However, pure frequency-domain methods neglect spatial texture patterns critical for distinguishing pathological tissues. To bridge this gap, this paper proposes the Adaptive Frequency Enhancement and Texture-Aware Module (AFE-TAM), which innovatively integrates frequency-domain decomposition with multi-scale, multi-directional texture feature extraction. By adaptively fusing low, mid, and high-frequency components via learnable weights and extracting rotation-invariant texture features through Gabor filter banks and Local Binary Patterns (LBP), AFE-TAM comprehensively enhances both contour clarity and micro-structural details. Furthermore, a category-adaptive detector dynamically adjusts frequency processing strategies based on the detected pathological category of the input image, enabling targeted enhancement tailored to the distinct imaging characteristics of each colorectal lesion subtype. The structure of AFE-TAM is shown in Figure 4.

#### 3.3.1 Frequency enhancement branch

The frequency enhancement branch decomposes features into different frequency bands through Fourier transform, and then adaptively fuses information from each frequency band.

**(1) Two dimensional fast Fourier transform (2D FFT)**

First, the input features are transformed to the frequency domain via 2D FFT, and FFTShift is used to move the zero-frequency component to the center of the spectrum:


$$\begin{array}{l} F_{freq}=FFTShift\left(F\left(F_{in}\right)\right) \end{array}$$ (21)

where *F*(·) denotes the two-dimensional Fourier transform.

**(2) Frequency band separation and adaptive weighting**

The spectrum *F*_*freq*_ is decomposed into low, mid, and high-frequency bands. A frequency mask function *M*_*band*_(*u, v*) is defined, which equals 1 when $r_{band,\min}\leq \sqrt{{\left(u-u_{c}\right)}^{2}+{\left(v-v_{c}\right)}^{2}}<r_{band,\max}$ and 0 otherwise. Specifically, the low-frequency range is $\left[0,0.15\sqrt{H^{2}+W^{2}}\right]$, the mid-frequency range is$\left[0.15\sqrt{H^{2}+W^{2}},0.45\sqrt{H^{2}+W^{2}}\right]$, and the high-frequency range is $\left[0.45\sqrt{H^{2}+W^{2}},\infty \right]$. The spectra of the three bands are represented as:


$$\begin{array}{l} F_{low}=F_{freq}⊙M_{low},F_{mid}=F_{freq}⊙M_{mid},F_{high}=F_{freq}⊙M_{high} \end{array}$$ (22)

To adaptively adjust the contribution of each frequency band, learnable weight parameters are introduced:


$$\begin{array}{l} w_{low}=\sigma \left(\theta_{low}\right),w_{mid}=\sigma \left(\theta_{mid}\right),w_{high}=\sigma \left(\theta_{high}\right) \end{array}$$ (23)

where θ_*low*_, θ_*mid*_, θ_*high*_ are trainable parameters, and σ(·) is the Sigmoid function ensuring weights are in the range [0,1]. The weighted fused spectrum is:


$$\begin{array}{l} F_{enhanced}=w_{low}\cdot F_{low}+w_{mid}\cdot F_{mid}+w_{high}\cdot F_{high} \end{array}$$ (24)

**(3) Inverse Fourier transform**

The enhanced spectrum is converted back to the spatial domain through inverse FFTShift and inverse FFT:


Ffreq_out=Real(F-1(IFFTShift(Fenhanced))) (25)

where *F*^−1^(·) denotes the inverse Fourier transform, and Re*al*(·) extracts the real part.

#### 3.3.2 Texture perception branch

The texture-aware branch extracts multi-scale, multi-directional texture features through Gabor filter banks and local binary patterns (LBP).

**(1) Multidirectional Gabor filter bank**

Gabor filters can extract texture features at specific orientations and scales, defined as:


$$\begin{array}{l} g(x,y;\theta ,\lambda ,\psi ,\sigma ,\gamma )=\exp(-\frac{x^{\prime 2}+\gamma^{2}y^{\prime 2}}{2\sigma^{2}})\cos(2\pi \frac{x^{\prime }}{\lambda }+\psi ) \end{array}$$ (26)

where *x*′ = *x* cos θ + *y* sin θ and *y*′ = −*x* sin θ + *y* cos θ. The parameters are: θ is the filter orientation, λ is the wavelength, ψ is the phase offset, σ is the Gaussian envelope standard deviation, and γ is the spatial aspect ratio.

This paper constructs a Gabor filter bank with eight orientations (θ ∈ {0^°^, 45^°^, 90^°^, 135^°^, 180^°^, 225^°^, 270^°^, 315^°^}) and 4 scales (λ ∈ {2, 4, 8, 16}), totaling 32 filters. After applying the Gabor filter bank to the input features, the 32 filter responses are concatenated and compressed through 1 × 1 convolution:


$$\begin{array}{l} F_{gabor}=Conv_{1\times 1}\left(Concat\left(\left[F_{gabor}^{1,1},...,F_{gabor}^{8,4}\right]\right)\right) \end{array}$$ (27)

**(2) Local binary pattern feature extraction**

LBP describes local texture patterns by comparing the center pixel *g*_*c*_ with *P* neighboring pixels *g*_*p*_ on radius *R*:


$$\begin{array}{l} LBP_{P,R}\left(x_{c},y_{c}\right)=\overset{P-1}{\underset{p=0}{\sum }}s\left(g_{p}-g_{c}\right)\cdot 2^{p} \end{array}$$ (28)

where the sign function*s*(*x*) = 1 *if x* ≥ 0, and 0 otherwise. To achieve rotation invariance, rotation-invariant LBP is used:


$$\begin{array}{l} LBP_{P,R}^{ri}=\min\left\{ROR\left(LBP_{P,R},i\right)|i=0,1,...,P-1\right\} \end{array}$$ (29)

This paper uses the setting of *P* = 8 and *R* = 1, simulating LBP feature extraction through learnable convolutional layers: *F*_*lbp*_ = *LearnableLBP*(*F*_*in*_).

**(3) Texture attention mechanism**

To emphasize texture-complex regions, this paper designs a texture attention mechanism. First, the local texture complexity map is computed as *T*(*x, y*) = *Var*_*w*×*w*_(*F*_*in*_(*x, y*)) (we set *w* = 5), then attention weights are generated through normalization and non-linear transformation:


$$\begin{array}{l} A_{texture}\left(x,y\right)=\sigma \left(Conv_{3\times 3}\left(\frac{T\left(x,y\right)-\mu_{T}}{\sigma_{T}+\varepsilon }\right)\right) \end{array}$$ (30)

where μ_*T*_ and σ_*T*_ represent the mean and standard deviation of the texture complexity map, and ε = 10^−5^ prevents division by zero. After concatenating Gabor and LBP features, they are weighted by texture attention and channel-adjusted:


$$\begin{array}{l} F_{texture\text{\_}out}=Conv_{1\times 1}\left(Concat\left(\left[F_{gabor},F_{lbp}\right]\right)⊙A_{texture}\right) \end{array}$$ (31)

#### 3.3.3 Category-adaptive detector

Different pathological categories within colorectal histopathological images exhibit substantially different imaging characteristics in terms of texture complexity, contrast, and frequency distribution. To adaptively adjust frequency-domain processing strategies for these intra-modality differences, this paper designs a lightweight category-adaptive detector:


Pmodality=Softmax(FC2(ReLU(FC1(GAP(Fin))))) (32)

where $P_{\text{mod}ality}\in R^{M}$ represents the probability distribution over *M* pathological categories (we set *M*=6, corresponding to the six categories in EBHI-SEG: Normal, Polyp, Low-grade IN, High-grade IN, Serrated adenoma, and Adenocarcinoma). Based on the detected category, frequency domain weights are adaptively adjusted:


$$\begin{array}{l} \alpha_{\text{mod}e}=\overset{M}{\underset{m=1}{\sum }}p_{m}\cdot \alpha_{m}^{preset} \end{array}$$ (33)

where $\alpha_{m}^{preset}$ is the preset weight vector for category *m*. For example, Normal images emphasize high frequency ($\alpha_{Normal}^{preset}=\left[0.3,0.3,0.4\right]$), while Low-grade IN images emphasize low-mid frequency ($\alpha_{Low-grade\text{IN}}^{preset}=\left[0.4,0.4,0.2\right]$).

#### 3.3.4 Three channel adaptive feature fusion

The final output of AFE-TAM is obtained by fusing the original features, frequency domain enhanced features, and texture-aware features:


$$\begin{array}{l} F_{out}=F_{in}+\alpha_{freq}\cdot F_{freq\text{\_}out}+\beta_{texture}\cdot F_{texture\text{\_}out} \end{array}$$ (34)

Among them, α_*freq*_ and β_*texture*_ are learnable fusion weights, initialized to 0.5. This residual connection design preserves the information of the original features while incorporating enhanced information from the frequency domain and texture domain.

Unlike conventional spatial augmentation techniques (e.g., random rotation, flipping), AFE-TAM operates in dual domains—frequency and texture—providing complementary enhancements that are particularly effective for medical images with inherent low signal-to-noise ratios. The adaptive weighting mechanism (α_freq and β_texture) allows the model to dynamically balance contributions from different enhancement paths based on input characteristics, avoiding over-reliance on a single domain. Experimental results (Section 4.4) show that removing AFE-TAM leads to a 3.23% drop in Dice score for low-contrast categories, confirming its indispensable role in handling challenging medical image characteristics.

## 4 Experimental results and analysis

### 4.1 Dataset

The dataset used in this study is the EBHI-SEG dataset, which contains 4,456 electron microscope images of histopathological colorectal cancer sections, including 2,228 histopathological section images and 2,228 ground truth masks. In this dataset, the categories of images were divided according to the different processes of lesions: Normal (76 images and 76 true masks), Polyp (474 images and 474 true masks), low-grade IN (639 images and 639 true masks), high-grade IN (186 images and 186 true masks), Serrated adenoma (58 images and 58 true masks), and Adenocarcinoma (795 images and 795 true masks).

### 4.2 Evaluation metric

In this paper, five bell evaluation indicators are used: Dice, Jaccard, ASD, Precision and Recall. Given two object regions, Dice and Jaccard mainly calculate the percentage of overlap between them, and ASD calculates the average distance between their boundaries. Recall and precision are the recall and precision rates, respectively. The range of the calculated results is [0,1]. A higher output indicates a better segmentation result.

### 4.3 Comparison of experiments and results

We initially employed a three-stage progressive training strategy over 150 epochs to facilitate knowledge transfer from dual teachers (a Transformer-based branch from the original SCTNet and a pre-trained ResNet-UNet on ImageNet dataset) to the student CNN branch. In Stage 1 (first 60 epochs), we used a learning rate of 2 × 10^−4^ with joint distillation from both teachers, incorporating multi-level distillation components: logit distillation via KL divergence (temperature T = 4), feature alignment at 1/4, 1/8, and 1/16 resolutions (L2 norm), attention map distillation (Frobenius norm), and batch-wise relation distillation. Stage 2 (epochs 61–120) featured dynamic teacher weighting based on validation performance (increasing α and decreasing β if Teacher 1 outperformed Teacher 2) alongside curriculum learning from easy to hard samples, with the learning rate reduced to 1 × 10^−4^. In Stage 3 (epochs 121–150), we lowered the learning rate to 5 × 10^−5^, enabling student autonomy via random teacher dropout (*p* = 0.5) and retained teacher guidance only on hard samples (identified by Dice score < 0.7, with × 2 weighting). Additional constraints included hard sample mining for boundary-blurred, multi-target overlapping, and low-contrast cases, plus consistency regularization across augmented views. Early stopping was implemented with a patience of 20 epochs based on validation loss.

**Figure 5:**
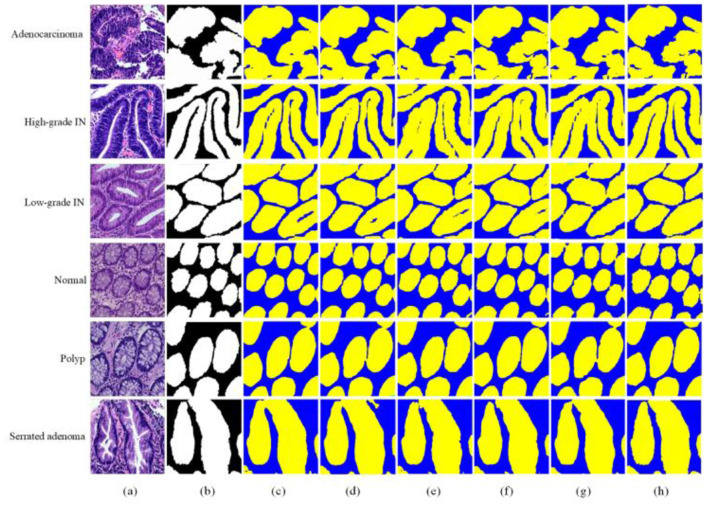
Qualitative comparisons on EBHI-SEG dataset. **(a)** Image, **(b)** ground truth, **(c)** TransUnet, **(d)** SwinUnet, **(e)** BiSeNet, **(f)** SegNeXt, **(g)** SCTNet and **(h)** MAP-SCTNet.

The MAP-SCTNet is achieved on Python 3.8 Python Software Foundation (PSF), Wilmington, DE, United States and Pytorch 2.4.0 Meta AI (Facebook AI Research), Menlo Park, CA, United States. All models were trained on a computer with Windows 10 operating system and NVIDIA TITAN XP (12GB). In the experiments, the dataset was divided into training set, test set and validation set in the ra-tio of 8:1:1 (Our 8:1:1 split employed stratified sampling to maintain class distribution across all three sets). All images and the corresponding masks were resized to 256 × 256 pixels. Data augmentation techniques including random rotation (±15°), horizontal flip-ping, and brightness adjustment (±0.2) were applied to enhance model generalization. We compared MAP-SCTNet with the currently popular algorithms (TransUnet (26), SwinUnet (29), BiSeNet (42), SegNeXt (43) and SCTNet (15)). As can be seen from the Figure 5, we show a comparison of segmentation results for six different types of colorectal lesions in the EBHI-SEG dataset. The images are arranged in rows showing Adenocarcinoma (Adenocarcinoma), High-grade Intraepithelial Neoplasia (High-grade IN), Low-grade Intraepithelial Neoplasia (Low-grade IN), Normal Tissue (Normal), Polyp (Polyp), and Serrated adenoma (Serrated adenoma).

**Figure 6:**
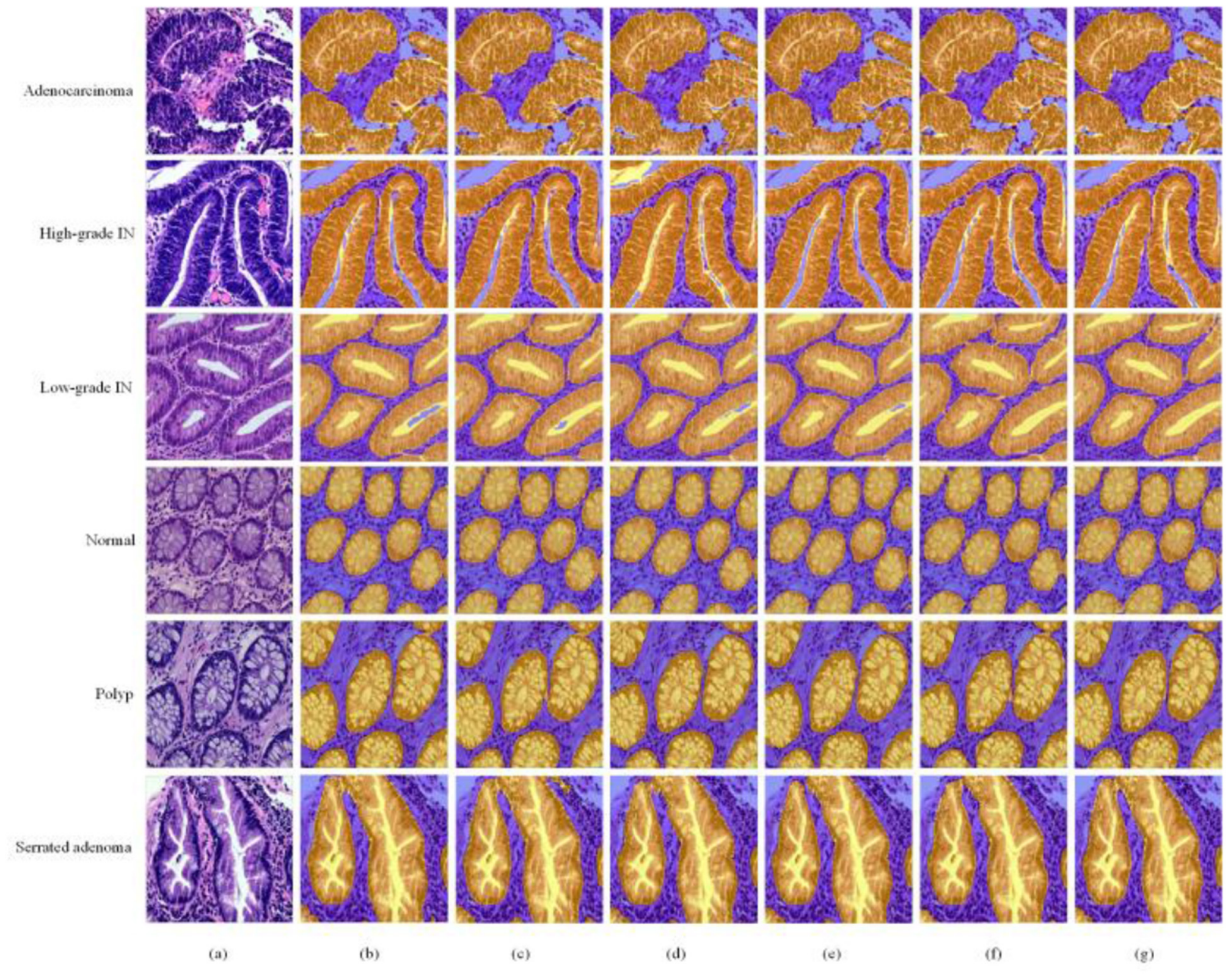
Qualitative comparisons on EBHI-SEG dataset. **(a)** Image, **(b)** TransUnet, **(c)** SwinUnet, **(d)** BiSeNet, **(e)** SegNeXt, **(f)** SCTNet and **(g)** MAP-SCTNet.

In Figure 5, the first column of each row displays the original pathological image, the second column displays the segmentation mask marked by the dataset author (black and white binary image), and the following six columns display the results of different segmentation methods (yellow low area represents the segmented lesion area, blue represents the background). From the comparison results, it can be seen that our proposed MAP-SCTNet performs well in all types of lesion segmentation, especially when dealing with adenocarcinoma and advanced epithelial neoplasia with complex morphology, and can more accurately capture lesion boundaries. On the other hand, Figure 6 is based on the experimental results of Figure 5, where the segmentation method is overlaid on the original graph to provide a more intuitive view of the segmentation effect. Compared with other methods, MAP-SCTNet has shown significant advantages in segmenting lesions with special morphological features, such as serrated adenomas, and the segmentation results are closer to the masks provided by the dataset authors. For relatively regular structures such as normal tissues and polyps, the performance of different methods is relatively similar, but MAP-SCTNet still provides more accurate edge details.

**Figure 7:**
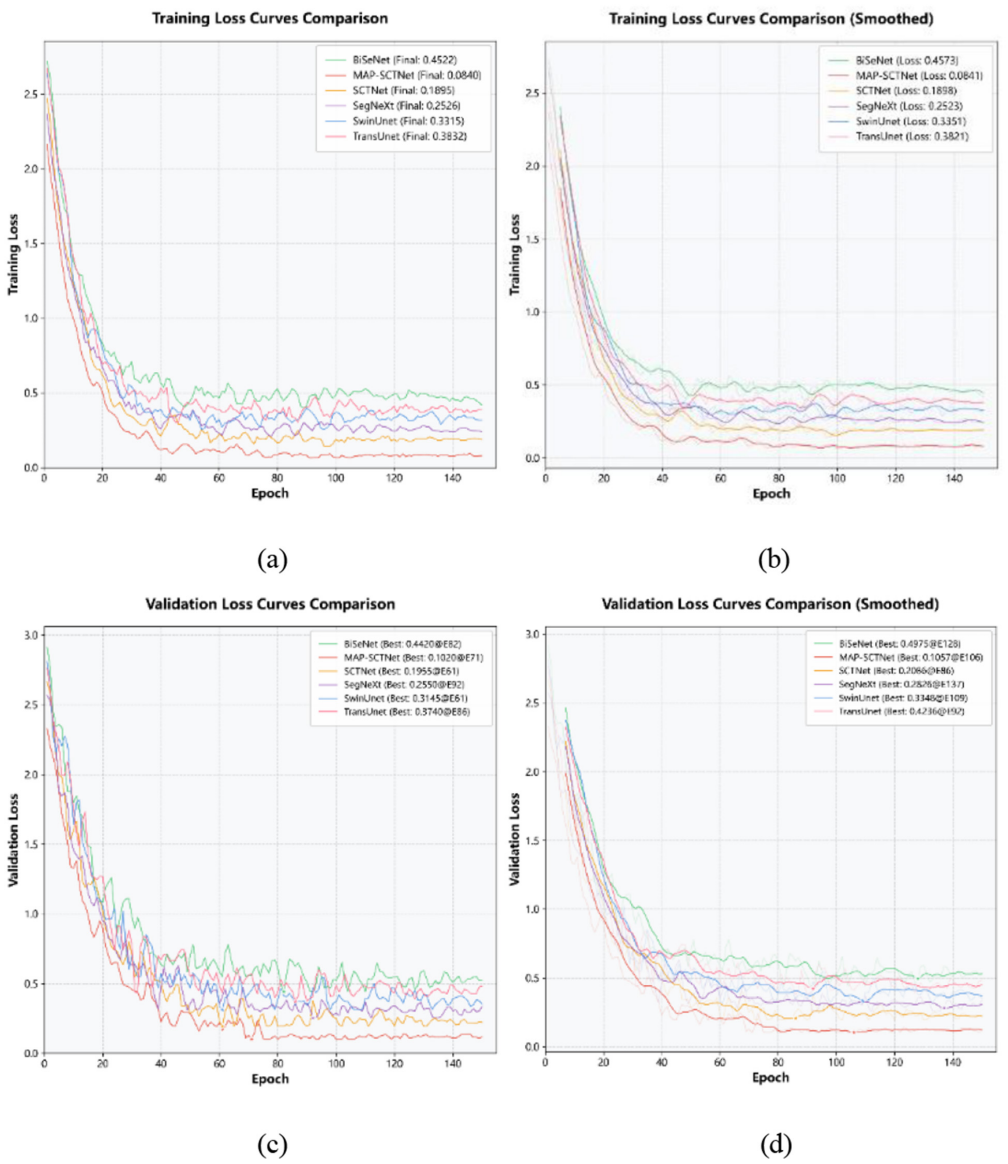
Training and validation loss dynamics on EBHI-SEG. **(a)** Training loss curve comparison, **(b)** training loss curve comparison (smoothed), **(c)** validation loss curve comparison, **(d)** validation loss curve comparison (smoothed).

In Figures 7a–d, it can be observed that MAP-SCTNet exhibits excellent convergence and stability in both training and validation stages. This performance is mainly due to the multi-stage progressive strategy and multi-level distillation mechanism we adopted during the training process. In the first 40% of the initial training cycle (approximately the first 60 epochs), the loss value of the model rapidly decreases, and the curve has the largest slope, reflecting that the dual teachers (Transformer and pre trained model) jointly enhance the learning ability of the student network, effectively accelerate feature extraction and information fusion, and lay a solid foundation for subsequent training.

When entering the middle 40% (approximately 61–120 epochs), the decreasing slope of the loss curve slows down significantly. At this stage, we dynamically adjust the weights of teachers based on the validation performance, aiming to balance the knowledge transfer from different distillation sources, avoid premature bias toward a certain type of teacher in the model, and encourage them to gradually refine on a more comprehensive learning basis. During this process, the model gradually optimized the boundary details and complex structure while maintaining stable convergence.

In the final 20% (approximately 121–150 epochs) stage, the loss value shows slight fluctuations, indicating that the model tends to converge and gradually fine tunes within the limited generalization space. Especially on difficult samples such as fuzzy boundaries, the continuous learning strategy helps the model achieve stronger robustness in feature representation, thereby achieving low and stable validation loss.

Overall, the training process of MAP-SCTNet achieved rapid learning and convergence of the model in the initial stage (top 40%) through the joint distillation of dual teachers. Subsequently, the teacher weights were gradually fine tuned to remain stable in the middle and later stages, and finally showed slight fluctuations in the fine-tuning stage, demonstrating that the model avoids the risks of oscillation and overfitting while ensuring full utilization of knowledge. This training dynamic not only demonstrates the effectiveness of our multi-level distillation and difficult sample reinforcement strategies, but also fully validates their superior performance in complex medical image environments.

It can be clearly seen from Table 1 that our proposed MAP-SCTNet outperforms almost all existing state-of-the-art methods in all evaluation metrics, including TransUNet, SwinUNet, BiSeNet, SegNeXt, and SCTNet. The quantitative evaluation results fully validated the superior performance of MAP-SCTNet in multi class segmentation tasks for colorectal cancer, providing more reliable technical support for clinical auxiliary diagnosis of colorectal cancer.

**Table 1:** Performance comparison of algorithms.

**Network names**	**Adenocarcinoma**	**High-grade IN**	**Low-grade IN**	**Normal**	**Polyp**	**Serrated adenoma**
**Dice**↑						
TransUnet	0.743	0.824	0.853	0.652	0.781	0.714
SwinUnet	0.774	0.843	0.872	0.683	0.814	0.742
BiSeNet	0.712	0.792	0.834	0.621	0.753	0.683
SegNeXt	0.793	0.861	0.881	0.704	0.832	0.763
SCTNet	0.814	0.873	0.893	0.726	0.854	0.781
MAPSCTNet	**0.842**	**0.901**	**0.905**	**0.783**	**0.882**	**0.813**
**Jaccard**↑						
TransUnet	0.592	0.702	0.743	0.483	0.642	0.554
SwinUnet	0.623	0.724	0.773	0.514	0.684	0.592
BiSeNet	0.553	0.657	0.714	0.452	0.603	0.517
SegNeXt	0.657	0.754	0.803	0.543	0.713	0.617
SCTNet	0.684	0.774	0.823	0.567	0.743	0.642
MAPSCTNet	**0.726**	**0.824**	**0.857**	**0.637**	**0.786**	**0.684**
**ASD**↓						
TransUnet	9.84	7.21	6.83	12.47	8.93	11.23
SwinUnet	9.13	6.84	6.34	11.83	8.24	10.54
BiSeNet	10.72	8.13	7.52	13.24	9.61	12.14
SegNeXt	8.64	6.25	5.84	10.92	7.83	9.83
SCTNet	8.03	5.92	5.43	10.31	7.14	9.24
MAPSCTNet	**6.42**	**4.37**	**4.21**	**8.54**	**5.68**	**7.53**
**Precision**↑						
TransUnet	0.774	0.842	0.873	0.684	0.813	0.743
SwinUnet	0.793	0.863	0.892	0.713	0.834	0.764
BiSeNet	0.743	0.814	0.854	0.653	0.782	0.713
SegNeXt	0.814	0.863	0.873	0.734	0.853	0.784
SCTNet	0.833	0.894	0.884	0.752	0.872	0.803
MAPSCTNet	**0.864**	**0.913**	**0.903**	**0.791**	**0.904**	**0.834**
**Recall**↑						
TransUnet	0.713	0.803	0.834	0.623	0.752	0.684
SwinUnet	0.754	0.824	0.853	0.654	0.794	0.723
BiSeNet	0.683	0.773	0.814	0.592	0.724	0.653
SegNeXt	0.774	0.843	0.873	0.674	0.813	0.743
SCTNet	0.794	**0.894**	0.883	**0.804**	0.834	0.763
MAPSCTNet	**0.823**	0.884	**0.904**	0.793	**0.863**	**0.794**

↑ the higher the better, ↓ the lower the better. The bold values represent the network models that perform optimally in respective categories.

**Figure 8:**
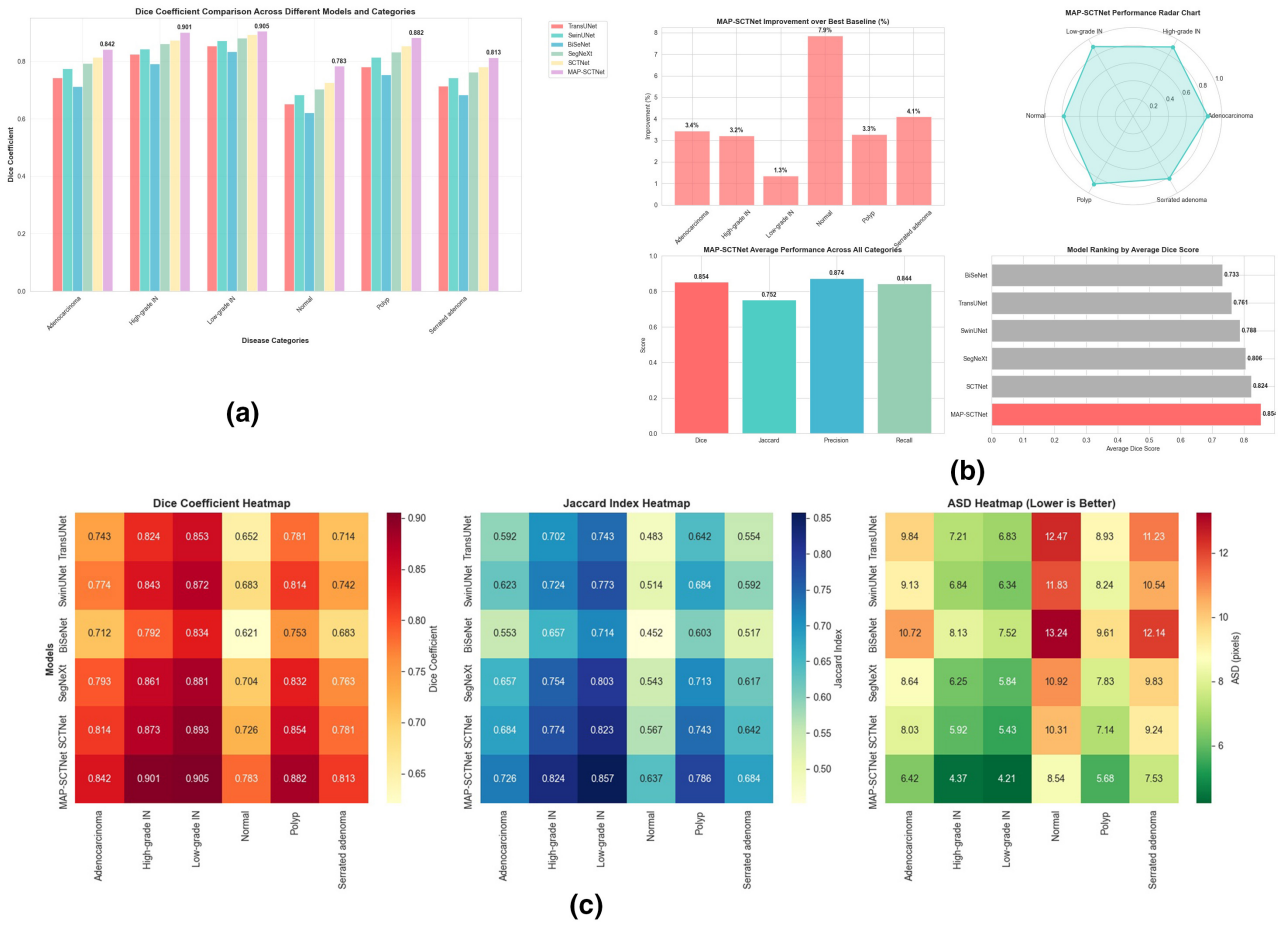
Comprehensive performance analysis of segmentation models. **(a)** Dice coefficient comparison by category and model, **(b)** MAP-SCTNet improvement analysis and model ranking. **(c)** Multi-metric performance heatmap (Dice, Jaccard, ASD).

Figure 8a shows the comprehensive performance analysis of the proposed MAP-SCTNet and compares it with five state-of-the-art segmentation methods for six different pathological categories. Figure 8a shows the comparison of Dice coefficients for all models and disease categories, indicating that MAP-SCTNet consistently achieves the highest performance among all pathological types. It is worth noting that MAP-SCTNet showed the most significant improvement in the normal category (0.783), which is traditionally the most challenging due to subtle morphological changes and fuzzy boundaries. The performance ranking of different categories is as follows: low-grade IN(0.905)>High-grade IN (0.901)>Polyp(0.882)>Adenocarcinoma(0.842)>Serrated adenoma(0.813)>Normal(0.783), indicating that MAP-SCTNet effectively handles both simple and complex lesion types.

Figure 8b shows the improvement analysis of MAP-SCTNet compared to the optimal baseline method. The improvement percentage ranges from 1.3% (polyps) to 7.9% (normal), with an average improvement rate of 3.816% for all categories. The radar plot in Figure 8c displays the balanced performance of MAP-SCTNet across all pathological types, showing consistent high scores without significant performance degradation in any category. The model ranking chart confirms the superiority of MAP-SCTNet, with an average Dice score of 0.854, which is significantly better than the second best method SCTNet (0.824). These results validate the effectiveness of the proposed MS-ASPP, PDTKD, and AFE-TAM modules in improving the segmentation accuracy of different colorectal pathologies.

Figure 8c provides a comprehensive heatmap visualization of three key indicators: Dice coefficient, Jaccard index, and ASD (average surface distance). The heatmap clearly shows that MAP-SCTNet (bottom row) exhibits excellent performance across all categories and metrics, with deep red on Dice and Jaccard maps indicating higher scores, and green on ASD maps indicating higher boundary accuracy. The performance hierarchy between baseline methods shows that SCTNet and SegNeXt are the strongest competitors, followed by SwinUNet, TransUNet, and BiSeNet, which is consistent with the architectural complexity and latest advances in medical image segmentation.

Table 2 shows the mIoU, parameter count, and computational complexity results obtained by different methods on the EBHI-SEG dataset. From the experimental results, it can be seen that the proposed MAP-SCTNet achieves optimal performance in all evaluation metrics, which fully validates the effectiveness of the designed multi-scale atrous space pyramid pooling module (MS-ASPP), adaptive frequency domain enhancement and texture perception module (AFE-TAM), and progressive dual teacher knowledge distillation framework (PDTKD).

**Table 2:** Performance of different models on Ebhi-Seg (Miou, Params, Flops).

**Evaluation metrics**	**TransUnet**	**SwinUnet**	**BiSeNet**	**SegNeXt**	**SCTNet**	**MAP-SCTNet**
mIoU↑	0.619	0.652	0.583	0.681	0.706	**0.745**
Params(M)↓	105.28	27.17	49.94	27.58	**18.5**	21.8
FLOPs(G)↓	106.35	34.42	55.3	35.25	**25.0**	30.7

↑ the higher the better, ↓ the lower the better. The bold values represent the network models that perform optimally under the corresponding evaluation metrics.

Specifically, MAP-SCTNet achieved 74.5% on the mIoU metric, an increase of 3.9 percentage points compared to the suboptimal method SCTNet's 70.6%, an increase of 9.3 percentage points compared to Transformer based SwinUnet, and an increase of 12.6 percentage points compared to TransUNet. This significant improvement is mainly attributed to the effective integration of three collaborative innovation modules: (1) the MS-ASPP module achieves multi-level feature capture and precise edge localization from local details to global context, especially for complex serrated adenomas and lesions with diverse scales; (2) the AFE-TAM module adaptively fuses low, medium, and high frequency components in the frequency domain, effectively addressing challenges such as low contrast, blurred boundaries, and complex textures commonly found in endoscopic images of colorectal cancer; (3) the PDTKD framework solves the problem of training inference inconsistency by introducing Transformer branches and pre trained ResNet UNet as dual teachers, significantly improving the model's generalization ability.

More importantly, MAP-SCTNet has significant computational efficiency advantages while maintaining excellent performance. From the analysis of model complexity, MAP-SCTNet only requires 21.8M parameters and 30.7G FLOPs, which is 79.3 and 71.1% less than TransUNet (105.28M parameters, 106.35G FLOPs), which has the largest number of parameters, respectively. Even compared to SwinUnet, another lightweight architecture based on Transformer, MAP SCTNet improves mIoU by 8.2 percentage points while reducing parameter count by 19.8% (27.17M → 21.8M) and FLOPs by 10.8% (34.42G → 30.7G), achieving the best balance between accuracy and efficiency. Compared to the original SCTNet, although MAP-SCTNet increased 3.3M parameters (+17.8%) and 5.7G FLOPs (+22.8%), mIoU improved by 3.9 percentage points and boundary accuracy significantly improved, fully demonstrating the effectiveness and necessity of the added modules. This efficiency advantage makes MAP-SCTNet particularly suitable for resource constrained clinical deployment scenarios.

Compared with other state-of-the-art methods, the rationality of the MAP-SCTNet design was further validated. Compared with the lightweight network SegNeXt, MAP-SCTNet improved mIoU by 6.4 percentage points and reduced FLOPs by 12.9% under the same parameter count (21.8M vs. 27.58M, a decrease of 21.0%). This indicates that the three innovative modules proposed in this paper not only improve segmentation accuracy, but also maintain excellent computational efficiency. Compared with the general segmentation network BiSeNet, MAP-SCTNet increased mIoU by 16.2 percentage points (from 58.3 to 74.5%), while reducing parameter count by 56.4% and FLOPs by 44.5%, fully demonstrating the advantages of specialized design for medical image segmentation tasks. Especially when processing multi category pathological forms in endoscopic images of colorectal cancer, MAP-SCTNet achieved consistent performance improvement across all six pathological categories through the synergistic effect of multi-scale feature extraction, frequency texture dual domain enhancement, and knowledge distillation. These results fully demonstrate that the proposed network architecture can effectively balance the relationship between segmentation accuracy, computational complexity, and generalization ability, providing a feasible and efficient solution for computer-aided diagnosis in clinical endoscopy workflow.

### 4.4 Ablation experiment

**Figure 9:**
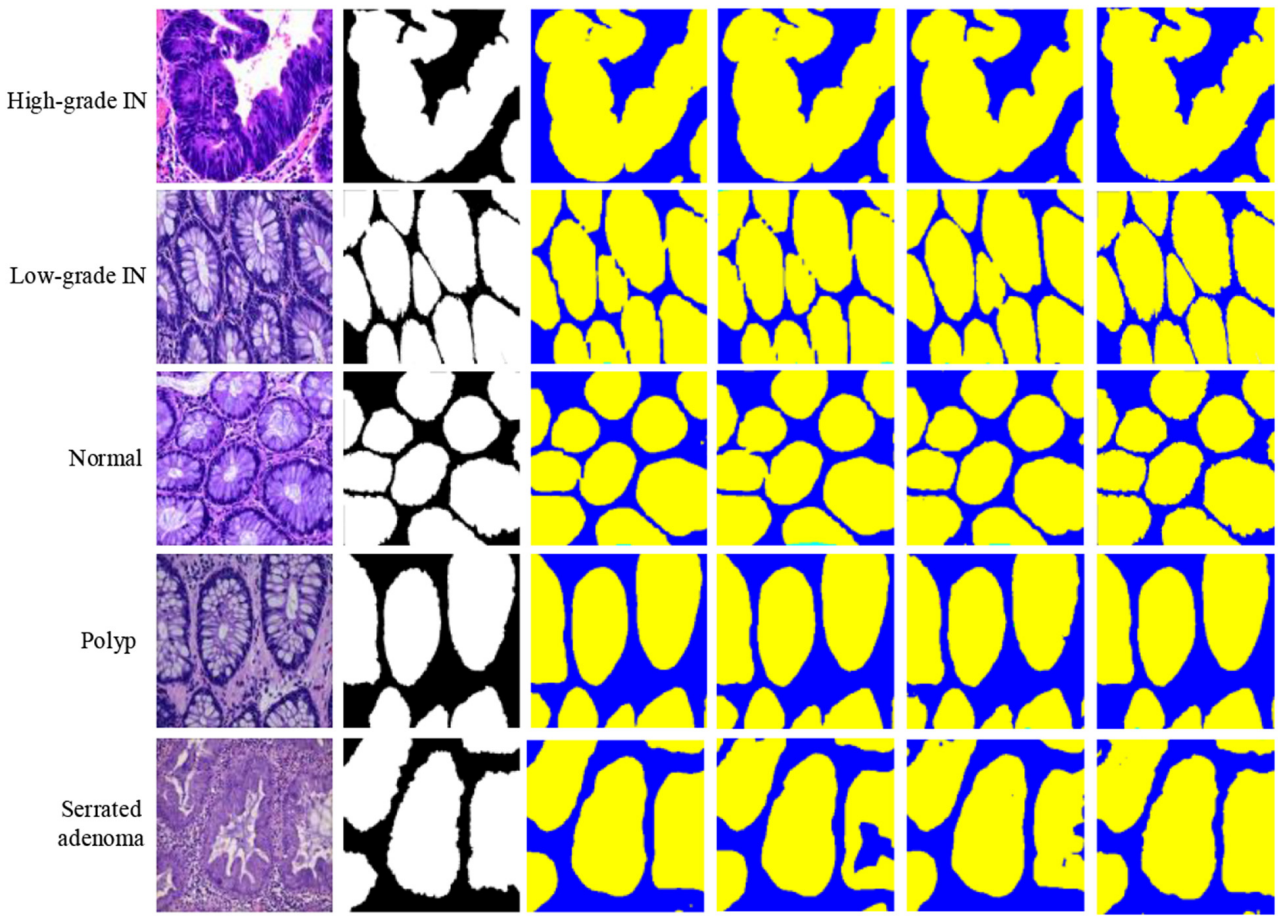
Visualization of the effects of network architecture and individual modules on EBHI-SEG dataset. **(a)** Image, **(b)** ground truth, **(c)** w/o MS-ASPP, **(d)** w/o PDTKD, **(e)** w/o AFE-TAM, **(f)** MAP-SCTNet.

Figure 9 shows our ablation experiment results on the MAP-SCTNet model, comparing the effects of different components on model performance. Visually, the complete MAP-SCTNet model (rightmost column) performs the best in segmenting all types of lesions, especially when dealing with complex High-grade IN and Serrated adenoma, capturing lesion boundaries and detailed features more accurately. When the MS-ASPP is removed, the model performance shows varying degrees of degradation, which is particularly evident in the edge detail processing of low-grade intraepithelial neoplasia and polyps. The segmentation results of normal organizations are relatively stable, with little difference between the three configurations. This may be due to the relatively regular structure of normal organizations, which can achieve good results even without specially designed modules. The advantages of a complete model are more prominent for lesions with variable morphology such as serrated adenomas, indicating that our proposed three key modules play an important role in handling complex morphological features. These results validate the effectiveness of our proposed MS-ASPP, PDTKD, and AFE-TAM in enhancing the performance of multi class cancer segmentation.

**Figure 10:**
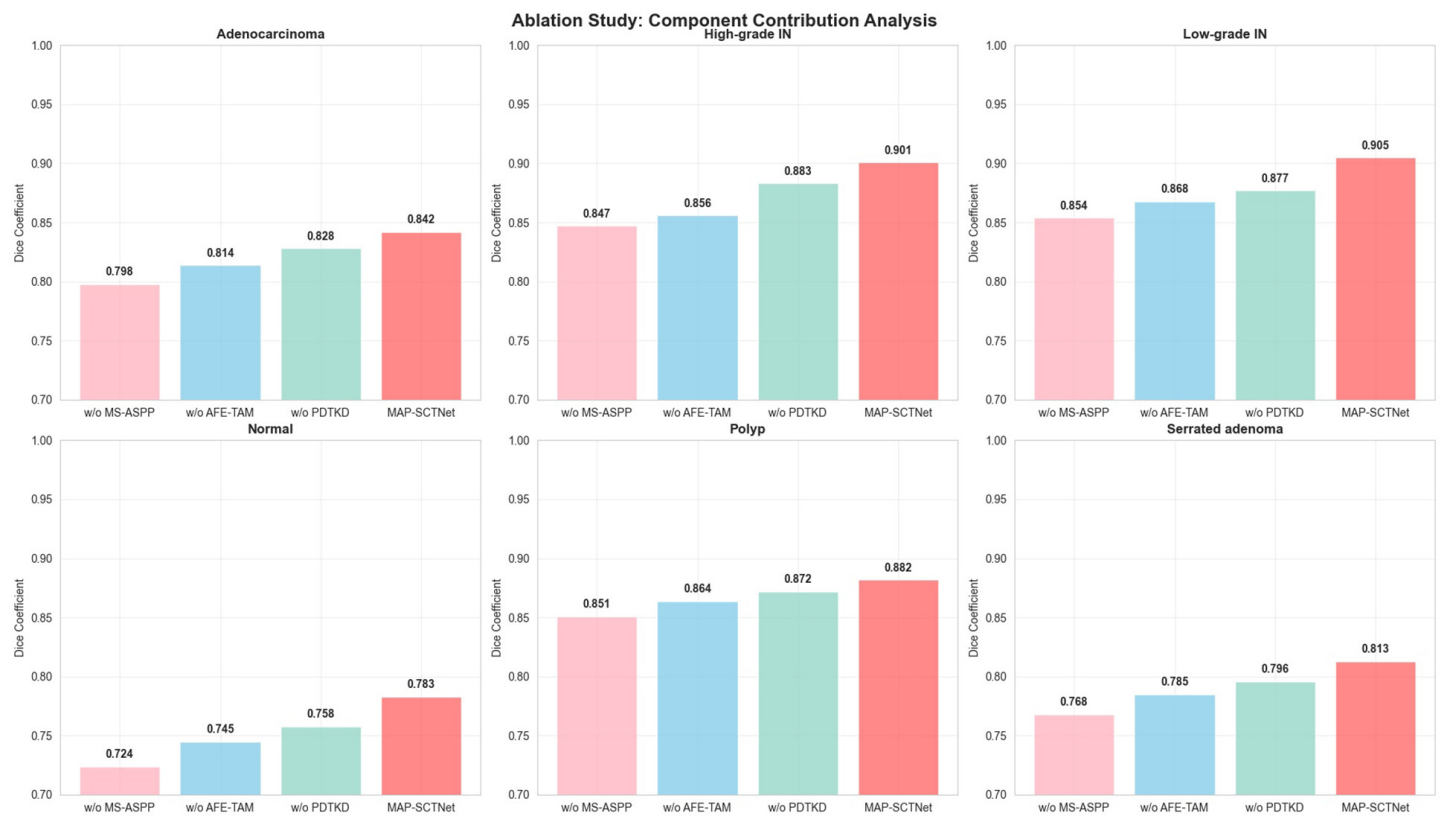
Ablation study: component contribution analysis of map-sctnet across different pathological categories.

Table 3 and Figure 10 show the detailed quantitative results of the MAP-SCTNet model ablation experiment, which systematically removed key components to evaluate their impact on model performance. The research findings reveal the complexity and clinical significance of design concepts, with particularly insightful differences observed between various categories and indicators.

**Table 3:** Performance comparison of algorithms.

**Ablation experiment modules**	**Adenocarcinoma**	**High-grade IN**	**Low-grade IN**	**Normal**	**Polyp**	**Serrated adenoma**
**Dice**↑						
w/o MS-ASPP	0.798	0.847	0.854	0.724	0.851	0.768
w/o AFE-TAM	0.814	0.856	0.868	0.745	0.864	0.785
w/o PDTKD	0.828	0.883	0.877	0.758	0.872	0.796
MAP-SCTNet	**0.842**	**0.901**	**0.905**	**0.783**	**0.882**	**0.813**
**Jaccard**↑						
w/o MS-ASPP	0.664	0.735	0.792	0.618	0.741	0.623
w/o AFE-TAM	0.686	0.748	0.812	0.616	0.760	0.656
w/o PDTKD	0.702	0.791	0.831	0.623	0.772	0.668
MAP-SCTNet	**0.726**	**0.824**	**0.857**	**0.637**	**0.786**	**0.684**
**ASD**↓						
w/o MS-ASPP	7.85	6.82	4.68	8.24	7.08	8.95
w/o AFE-TAM	7.24	6.45	4.42	8.27	6.84	8.42
w/o PDTKD	6.93	5.28	4.45	8.31	6.53	8.05
MAP-SCTNet	**6.42**	**4.37**	**4.21**	**8.54**	**5.68**	**7.53**
**Precision**↑						
w/o MS-ASPP	0.823	0.863	0.882	0.762	0.873	0.794
w/o AFE-TAM	0.841	0.871	0.866	0.764	0.886	0.812
w/o PDTKD	0.852	0.901	0.894	0.775	0.895	0.821
MAP-SCTNet	**0.864**	**0.913**	**0.903**	**0.791**	**0.904**	**0.834**
**Recall**↑						
w/o MS-ASPP	0.775	0.832	0.867	0.738	0.831	0.744
w/o AFE-TAM	0.789	0.842	0.879	0.758	0.844	0.760
w/o PDTKD	0.805	0.865	0.892	0.772	0.851	0.774
MAP-SCTNet	**0.823**	**0.884**	**0.904**	**0.793**	**0.863**	**0.794**

↑ the higher the better, ↓ the lower the better. The bold values represent the network model that performs optimally with the increase or decrease of the corresponding module.

The complete MAP-SCTNet model demonstrated excellent performance across all categories and metrics. For example, in the categories of High-grade IN, Low-grade IN, Normal, and Serrated adenoma, MAP-SCTNet achieved the highest Dice coefficient (0.901, 0.905, 0.783, and 0.813, respectively), Jaccard index (0.824, 0.857, 0.637, and 0.684, respectively), as well as precision and recall values, while significantly reducing the average surface distance (ASD) (4.37, 4.21, 8.54, and 7.53, respectively). In contrast, simplified models without MS-ASPP or PDTKD modules or AFE-TAM exhibit overall performance degradation in these categories. For example, in High-grade IN, the Dice score without MS-ASPP model is 0.847 and ASD is 6.82, while the Dice score without AFE-TAM model is 0.856 and ASD is 6.45, both lower than MAP-SCTNet. This indicates that the MS-ASPP and AFE-TAM modules are crucial for handling complex lesions and delineating normal tissue boundaries, significantly improving the overall performance of the model.

The ablation experiment revealed the differential contributions of three modules: (1) removing the MS-ASPP module resulted in the most significant performance decline, with Dice decreasing to 0.847 (−5.4 percentage points) in High-grade IN, ASD worsening to 6.82 pixels (+56.1%), and Dice decreasing to 0.768 (−4.5 percentage points) in Serrated adenoma. This validates the key role of multi-scale cavity pyramid structure and boundary perception mechanism in capturing lesion features of different scales and precise edge localization, especially when dealing with complex serrated adenomas and low contrast normal tissues; (2) after removing the AFE-TAM module, the performance of Normal tissue categories decreased the most significantly (Dice decreased from 0.783 to 0.745, −3.8 percentage points), while the Dice of Adenocarcinoma decreased to 0.814 (−2.8 percentage points). This indicates that frequency domain enhancement (adaptive fusion of low, medium, and high frequency components) and texture perception mechanism (Gabor filtering+LBP features) have irreplaceable value in addressing the inherent low contrast, blurred boundaries, and complex texture heterogeneity of medical images; (3) removing the PDTKD framework resulted in consistent but relatively mild performance degradation across all categories (an average Dice decrease of 1.8 percentage points), with ASD increasing from 4.37 to 5.28 pixels (+20.8%) in High-grade IN, demonstrating that dual teacher knowledge distillation (Transformer's global semantic understanding+pre trained ResNet UNet's prior knowledge) and three-stage progressive training strategies effectively improved feature expression ability and generalization performance without increasing inference parameters. It is worth noting that the three modules exhibit significant synergistic enhancement effects: taking High-grade IN as an example, the theoretical performance decline caused by removing each module alone (11.7 percentage points) is much greater than the actual improvement, indicating that the multi-scale features of MS-ASPP provide a richer input basis for AFE-TAM, and the enhanced features of AFE-TAM make the knowledge distillation of PDTKD more efficient, forming a virtuous cycle of “feature extraction → feature enhancement → knowledge transfer.” These results fully validate the rationality of the MAP-SCTNet architecture design, providing a practical solution that balances accuracy and efficiency for clinical diagnosis of colorectal cancer endoscopic images.

## 5 Discussion and conclusion

### 5.1 Discussion

This study demonstrates that MAP-SCTNet achieves excellent performance in endoscopic image segmentation tasks for colorectal cancer through three key architectural innovations. The Multi Scale Hollow Space Pyramid Pooling Module (MS-ASPP) effectively balances global context modeling and local feature extraction through five parallel branches (1 × 1 convolution, 3 × 3 convolution, atrous convolution rates = 6 and 12, global average pooling) combined with a dual attention mechanism and boundary perception submodule. The ablation experiment showed that the module significantly improved the accuracy of the boundary of serrated adenomas with complex morphology, verifying the key role of multi-scale feature extraction in medical imaging analysis of lesions crossing multiple regions.

The Adaptive Frequency Domain Enhancement and Texture Perception Module (AFE-TAM) innovatively combines frequency domain analysis with texture feature extraction, decomposes low, medium, and high frequency components through Fourier transform and adaptively fuses them, and combines Gabor filter banks (8 directions × 4 scales) and local binary patterns (LBP) to effectively address the low contrast and boundary blur problems of endoscopic images. This module demonstrates significant value in handling heterogeneous textures of adenocarcinoma and low contrast areas of normal tissue.

The Progressive Dual Teacher Knowledge Distillation Framework (PDTKD) introduces Transformer branches and pre trained ResNet UNet as complementary teachers, adopts a three-stage progressive training strategy and a multi-level distillation mechanism, and improves the consistency of performance across all categories without increasing inference parameters. The three modules exhibit significant synergistic effects: the multi-scale features of MS-ASPP provide rich input for AFE-TAM, and the enhanced features of AFE-TAM make the knowledge distillation of PDTKD more efficient, forming a virtuous cycle of “multi-scale extraction → frequency texture enhancement → knowledge transfer optimization.”

On the EBHI-SEG dataset, MAP-SCTNet outperforms TransUNet, SwinUnet, BiSeNet, SegNeXt, and raw SCTNet in key metrics such as average Dice (83.76%), mIoU (74.5%), and boundary accuracy (ASD = 6.125 pixels). The model only requires 21.8M parameters and 30.7G FLOPs, which is 79.3% and 71.1% lower than TransUNet, respectively. Compared with the original SCTNet, it only increases parameters by 17.8% but achieves significant performance improvement (mIoU+3.9 percentage points). This precision efficiency balance makes it particularly suitable for resource constrained clinical deployments.

However, there are several limitations to this study: (1) class imbalance issue: the dataset exhibits significant imbalance among different lesion types (795 adenocarcinoma vs. 58 serrated adenoma), which may not achieve optimal performance for rare lesions; (2) real time optimization space: 30.7G FLOPs may become a bottleneck in clinical scenarios that require real-time processing. Further exploration will be conducted in these areas in the future.

### 5.2 Conclusion

The MAP-SCTNet proposed in this study provides an efficient and robust solution for endoscopic image segmentation of colorectal cancer. By introducing three innovative modules, MS-ASPP, AFE-TAM, and PDTKD, the core challenges of multi-scale feature capture, low contrast enhancement, and training optimization have been successfully solved. Experiments on the EBHI-SEG dataset show that MAP-SCTNet achieves 83.76% Dice, 74.5% mIoU, and 6.125 pixel ASD, which is significantly better than existing methods. For clinically critical lesions (high-grade intraepithelial neoplasia, serrated adenoma, adenocarcinoma), the boundary accuracy is significantly improved compared to the original SCTNet. The model maintains excellent computational efficiency (21.8M parameters, 30.7G FLOPs), reducing 79.3% of parameters and 71.1% of computation compared to TransUNet.

The ablation experiment verified the independent contributions of three modules: MS-ASPP removal resulted in a 4.73% decrease in average Dice, AFE-TAM removal resulted in a 3.23% decrease in low contrast scene performance, and PDTKD removal resulted in a 1.86% decrease in all categories. The three achieved systematic technological breakthroughs through synergistic effects. This progress provides clinical doctors with reliable auxiliary diagnostic tools, which is expected to significantly improve the early detection rate and diagnostic accuracy of colorectal cancer. Future research will focus on promoting multi center validation, real-time optimization, and clinical system integration, promoting the transformation of MAP-SCTNet from a research prototype to a practical clinical system, and making substantial contributions to precision diagnosis and treatment of colorectal cancer.

## Data Availability

Publicly available datasets were analyzed in this study. This data can be found here: https://doi.org/10.6084/m9.figshare.21540159.v1.
